# Sex differences in blood pro-oxidant status and platelet activation in children admitted with respiratory syncytial virus bronchiolitis: a pilot study

**DOI:** 10.1186/s13052-020-0792-x

**Published:** 2020-03-06

**Authors:** Isabella Tarissi De Jacobis, Rosa Vona, Elisabetta Straface, Lucrezia Gambardella, Giulia Ceglie, Francesca de Gennaro, Ilenia Pontini, Anna Chiara Vittucci, Alessandra Carè, Camilla Cittadini, Alberto Villani, Donatella Pietraforte

**Affiliations:** 1 Internal Care Department, General Pediatric and Infectious Disease Unit, Bambino Gesù Children’s Hospital, Rome, Italy; 2 Biomarkers Unit, Center for Gender-Specific Medicine, Istituto Superiore di Sanità, Viale Regina Elena 299, 00161 Rome, Italy; 3 Department of Pediatrics, Pediatric Infectious Diseases Unit, Bambino Gesù Children’s Hospital, Rome, Italy; 4 Core Facilities, Istituto Superiore di Sanità, Rome, Italy

**Keywords:** Bronchiolitis, Respiratory syncytial virus, Platelets, Reactive oxidizing species, Sex differences

## Abstract

**Background:**

Respiratory syncytial virus (RSV) is the most common cause of bronchiolitis in the pediatric population worldwide and an important cause of death in developing countries. It has been demonstrated that the balance between oxidant and antioxidant systems is disrupted in children with bronchiolitis and that oxidative stress contributes to the pathogenesis of this disease. Platelets play an important role in antimicrobial host defenses and contribute to pulmonary vascular repair being either targets or source of reactive oxidizing species. The main purpose of this study was to assessing sex differences in clinical characteristics and platelets activation during RSV bronchiolitis in infancy.

**Methods:**

In this retrospective study a total of 203 patients (112 boys and 91 girls) with bronchiolitis, aged 12 months or less, admitted to the Bambino Gesù Pediatric Hospital of Rome (Italy) in the period from January to December 2017, were enrolled. Moreover, in a select group of patients (15 boys and 12 girls) with diagnosis of moderate bronchiolitis from RSV, a pilot study on oxidative stress and platelet characteristics was carried out by electron paramagnetic resonance and flow cytometry respectively. Age-matched healthy control subjects (10 boys and 10 girls) were chosen as controls. Data were analyzed using Student’ T test, Chi Squared test and one-way ANOVA test.

**Results:**

This study highlights the influence of sex in the clinical course of bronchiolitis. In particular we found: i) a higher incidence of bronchiolitis in boys than in girls (55% vs 45%); ii) higher C reactive protein values in girls than boys (1.11 mg/dL vs 0.92 mg/dL respectively; *p* < 0.05); iii) a different degree of thrombocytosis during hospitalization (mild in the girls and severe in the boys). Moreover, in selected patients we found that compared to girls with bronchiolitis, boys showed: i) higher percentage of activated platelets (8% vs 2% respectively; *p* < 0.05) and iii) higher number of platelets forming homotypic aggregates (2.36% vs 0.84% respectively, *p* < 0.05).

**Conclusion:**

The present study affirm that the bronchiolitis is an infection in which sex seems to act as a modulating factor only in the clinical course, influencing also the choice of the therapy should be made.

## Background

Bronchiolitis is the result of common viral infection in young children, usually caused by respiratory syncytial virus (RSV) and rhinovirus. RSV frequently causes severe morbidity and mortality, especially in premature infants and children with other chronic diseases [1]. In particular, RSV infection in infants with bronchiolitis can be associated with a higher rate of thrombocytosis [2]. Platelets play an important role in antimicrobial host defense, induction of inflammation and tissue repair, but the mechanism utilized by infection to induce thrombocytosis in these patients has not yet been understood. Probably, numerous inflammatory cytokines, such as thrombopoietin, IL-6, Il-1α, IL-8, IL-11 and TNFα play a decisive role. Platelets also regulate pulmonary vascular permeability and influence pulmonary vessel-reactivity, and lungs are sites of thrombopoiesis (process of platelet production) [3].

Oxidative stress (OxS) could play an important role in the pathogenesis of moderate bronchiolitis [4]. Also platelets could contribute to systemic OxS being either targets or source of reactive oxidizing species (ROS), conditions that can dramatically affect platelet physiology leading even, as an ultimate event, to the cell number modification [5].

Sex has been reported to have a key role in the morbidity and mortality of infectious diseases [6]. Starting from the birth, boys indeed show increased susceptibility and severity of a range of infectious diseases, including RSV, adenovirus, mumps, measles, with respect to girls [6]. These differences have been attributed to sex hormones which impact on immune system, with girls showing a stronger humoral and cellular immune responses to antigenic stimulation or to infection [6].

The main purpose of this study was to determine the role of platelets during RSV infections and investigate whether sex can affect the clinical characteristics of children with bronchiolitis.

## Methods

### Patients selection

In this retrospective study a total of 388 patients, with a first episode of bronchiolitis, admitted to the Bambino Gesù Pediatric Hospital of Rome (Italy) in the period from January to December 2017, were enrolled. Of all the hospitalized children, we selected only those aged 12 months or less (age with higher incidence of bronchiolitis). We excluded from the study patients with pathologies or infections that could modify the clinical course of bronchiolitis worsening the prognosis. In particular, we excluded: 80 patients (21%) infected by unknown viruses; 45 patients (12%) with history of prematurity (less than 37 weeks); 40 patients (10%) with immunodeficiency; 20 patients (5%) with congenital heart diseases. The analysis was conducted on only 203 patients (52% of enrolled patients): 112 boys and 91 girls. For all patients, disease severity score was constructed based on the following variables: dyspnea, tachypnea, hypoxia (oxygen saturation < 92), cough, fever and length of hospitalization. For each symptom, the patient was “accredited” one point. Patients with the median score or above were classified as having a severe illness.

Based on the degree of bronchiolitis (moderate or severe) oxygen was administered with low or high flow through nasal tubes.

Virus infections were detected in nasopharyngeal secretions by real-time PCR (RT-PCR). The characteristics of all the patients included in the study are shown in Table 1. Based on the clinical differences highlighted on patients admitted from January to December 2017, we selected a group of patients admitted from January to March 2018 [7] to conduct a pilot study on OxS and platelet characteristics. In particular, a group of patients with diagnosis of moderate bronchiolitis (15 boys and 12 girls) was selected. The diagnosis of moderate bronchiolitis was performed according to Berardi et al., [8] (see Table 2). We chose patients with moderate bronchiolitis because in this form of bronchiolitis the oxygen support is delivered with low flow rates, while in patients with severe forms of bronchiolitis the oxygen support is delivered with high flows. Moreover, severe forms of bronchiolitis could alter the pro-oxidant state of the blood and the activation of the platelets in the two different sexes.

**Table 1:** Mean characteristics of patients admitted from January to December 2017

	Boys (*n* = 112)	Girls (*n* = 91)	*P* value
Age, months	2.4 (1–10)	3.3 (1–12)	ns
Hospitalization (days)	5.13 (6–19)	5.8 (1–19)	ns
Illness duration (days)	10.5 (6–20)	8.7 (3–19)	ns
CRP, mg/dL	0.92 (range 0.46–6.28)	**1.11** (range 0.36–14.32)	*P* < 0.05
RSVB (% of patients)	**58%**	47%	*P* = 0.030
RSVA (% of patients)	11.6%	16%	ns
Rhinovirus (% of patients)	6.25%	4%	ns
Metavirus	9.8%	7%	ns
Other viruses	7.1%	**19%**	*P* = 0.05
Coinfections (% of patients)	4.46%	7%	ns
Complications of the disease	12.5%	11%	ns
Mild thrombocytosis	78.3%	**90%**	*P* = 0.01
Severe thrombocytosis	**21.7%**	10%	*P* = 0.05
Oxygen therapy (% of patients)	42.86.%	48%	ns
Cortisone therapy (% of patients)	46.4%	**60%**	*P* = 0.05
Antibiotic therapy (% of patients)	35.7%	35%	ns
Aerosol therapy with 2 ml of 3% hypertonic solution (% of patients)	62%	54%	ns

*CRP* C reactive protein, *RSVA* Respiratory syncytial virus A, *RSVB* Respiratory syncytial virus B

**Table 2:** Characteristic of patients with moderate bronchiolitis

	Moderate bronchiolitis
Respiratory rate	Increased
Respiratory effort	Tracheal tug Nasal Flare Moderate chest wall retraction
Oxygen saturations	Saturation 90–95%
Feeding	50–75% of normal feeds
Apnoea	Brief episodes

This selected group of patients with moderate bronchiolitis was limited to patients infected by RSVB, that is the most common cause of bronchiolitis in both male and female infants and that frequently causes severe morbidity and mortality [1].

The analyses were carried out after the diagnosis of bronchiolitis (about 2 or 3 days after admission).

Age-matched healthy subjects (10 boys and 10 girls) were used as control group (HC). This group consisted of healthy children who have no clinical symptoms and that came to the hospital for a check-up after diarrhea, poor growth or dehydration.

In this study the use of healthy subjects excludes that data obtained in patients are not due to artifacts, but to the disease. Informed consent by parents of both patients and HCs was obtained. The study has been approved by the Institutional Review Board of the Bambino Gesù (approval number: 1489OPBG2017). The investigation conforms the principles outlined in the Declaration of Helsinki.

### Platelet isolation

Fresh whole blood samples were collected in acid-citrate- dextrose tubes (ACD; NIH formula A), and immediately centrifuged at 200 g for 12 min at room temperature to separate platelet-rich plasma (PRP). Additional ACD was added (one part ACD per three parts PRP) and platelets were pelleted at 800 g for 15 min as previously reported [9] for ROS measurement.

ROS released in fresh whole blood were measured by electron para-magnetic resonance (EPR) spin trapping technique as previously reported [10]. Briefly, the spin trap 1-Hydroxy-3-Carboxy-Pyrrolidine (CPH; ENZO Life Sciences, Farmingdale, NY) was incubated (1 mM) with fresh whole blood for 20 min at 37 °C. Samples were then drawn up into a gas-permeable Teflon tube and inserted into a quartz tube. EPR spectra were measured in air at 37 °C on a Bruker e-Scan (Bruker, Rheinstetten, Germany). The intensity of the CPH-derived characteristic 3-line spectrum attributable to the nitroxide 3-carboxyproxyl radical (CP^●^) (hyperfine coupling constant 1.63 ± 0.04 mT) was measured as peak-to-peak linewidth and taken as the measure of the totality of oxidants formed. Spectrometer conditions: modulation frequency, 100 kHz; microwave frequency, 9.4 GHz; microwave power, 20 mW; gain 1 × 10^4^; modulation amplitude, 0.1 mT; conversion time, 20.5 ms; time constant, 82 ms; sweep time, 21 s; number of scans, 1 [10].

### Platelet activation and platelet apoptosis

Platelet activation was assessed by flow cytometry using fluorescein Isothiocyanate-conjugated annexin V. Evaluation of apoptosis was performed by flow cytometry after double staining using fluorescein Isothiocyanate-conjugated annexin V (1: 200) and 0.05% trypan blue for 10 min at room temperature. All samples were analyzed by FACSCalibur cytometer (Becton Dickinson, Mountain View, CA, USA) in the FL1 and FL3 channels to determine the percentage of dead cells.

### Platelet adhesion

Heterotypic adhesion (platelets-lymphocytes) was evaluated by using monoclonal anti-CD62 IgG-Phycoerythrin-conjugated (1: 20; Becton Dickinson), while homotypic adhesion (platelets-platelets) was evaluated by using monoclonal anti PAC-1 IgG-FITC-conjugated (1: 20; Becton Dickinson). All samples were stained at 4 °C for 30 min with monoclonal antibodies and analyzed on a FACSCalibur cytometer (Becton Dickinson, Mountain View, CA, USA) equipped with a 488 nm argon laser. At least 20,000 events have been acquired.

### Statistical analyses

To compare average values of a continuous variable between two groups we used the Student’ T test, and to analyze relationship between two categorical variables we used Chi Squared test.

Cytofluorimetric results were analyzed by using the Kolmogorov–Smirnov test using Cell Quest Software. At least 20,000 events were acquired. The percentage of platelet positives to annexin V, CD62 and PAC-1 were used to provide a semi-quantitative analysis. Results are displayed as average value ± standard deviation, unless otherwise specified. Statistical analysis was performed by one-way analysis of variance (ANOVA). When a significant interaction was detected, we also performed a Bonferroni post-hoc test. *p* < 0.05 was considered as a threshold for a significant difference.

## Results

### Sex differences in clinical features of patients enrolled in the study

In patients admitted to the Bambino Gesù Pediatric Hospital of Rome (Italy) in the period from January to December 2017, some sex differences have been highlighted (Table 1).

The virus evaluation, performed in nasopharyngeal secretions of selected patients with bronchiolitis, allowed to revealed a sex-related distribution of two distinct groups: RSVA and RSVB. In particular, the most common virus detected in these patients was RSVB (in 58% of boys and in 47% of girls), followed by RSVA (in 11.6% of boys and in 16% of girls). Interestingly, significant (*p* = 0.030) sex difference has been found in RSVB infection.

Oxygen therapy was used, without significant difference, in 42.86% of boys and in 48% of girls.

No significant differences were also detected in the use of aerosol therapy (in 62% of boys and in 54% of girls). Contrarily, the use of cortisone was significantly different in the sexes. Indeed, cortisone therapy was used in 46.4% of boys and in 60% of girls (*p* = 0.05). However, we found that during the hospitalization patients developed mild thrombocytosis (78.3% of boys and 90% of girls; *p* = 0.01) and severe thrombocytosis (21.7% of boys and 10% of girls; *p* = 0.05) (Table 1). On a clinical level, thrombocytosis is classified “mild” at a platelet count between > 500.000 μL and < 700.000 μL and “severe” at a platelet count > 900.000/μL.

### EPR measurement of ROS in whole blood

Inflammation-promoted ROS overproduction not counteracted by an efficient intracellular antioxidant system is a condition known as OxS. It leads to the irreversible cellular damage due to the altered cellular trafficking and redox signaling, and is frequently associated with cardiovascular and neurodegenerative diseases such as atherosclerosis, hypertension, diabetes, pulmonary fibrosis, Alzheimer’s disease, as well as cancer. Since bronchiolitis is an inflammatory respiratory condition, we measured the ROS-mediated formation of CP^•^ in fresh whole blood of selected patients (admitted from January to March 2018) in comparison with that measured in sex-matched controls.

**Fig. 1:**
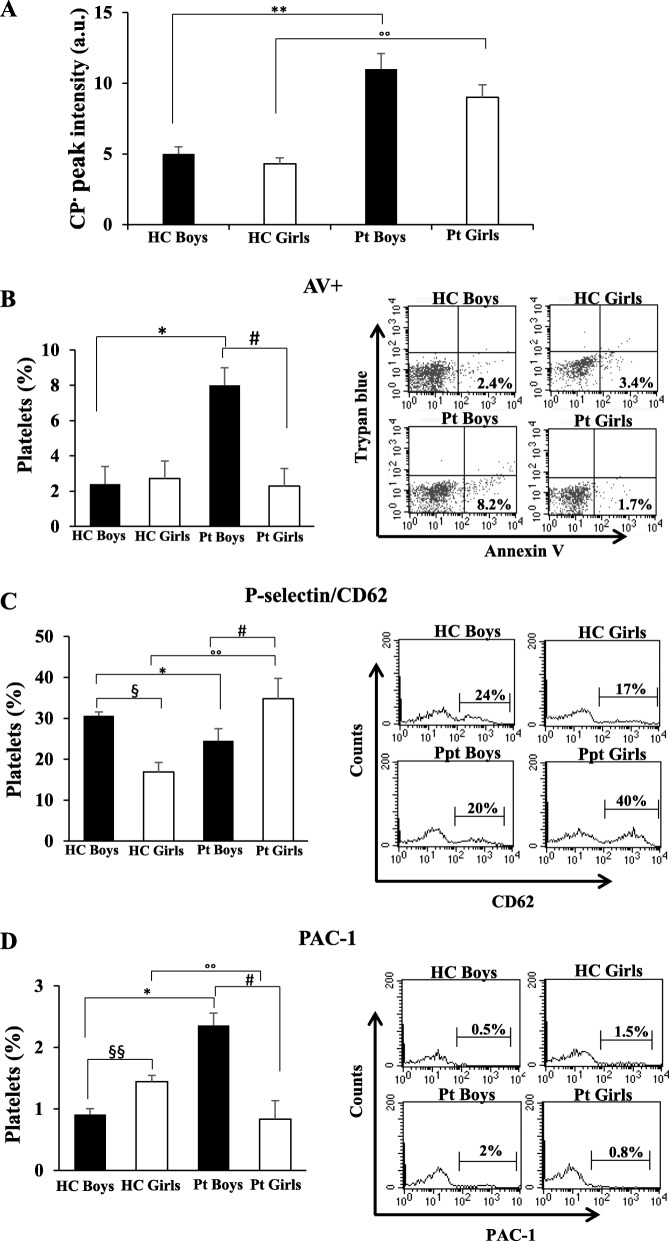
ROS production evaluation and platelet characteristics in patients with bronchiolitis (15 boys and 12 girls). **a** ROS levels measured in fresh whole blood by electron paramagnetic resonance. ##/°°*p* < 0.01 (Patients vs HCs). The measures were carried out in triplicate. Histograms showing the percentage of platelets positives to: **b** Annexin V; **c** P-selectin/CD62; **d** PAC-1. Measures carried out by flow cytometry. The values represent the percentage ± SD of positive cells. Statistical analysis was performed by one-way analysis of variance (ANOVA). # *p* < 0.05 (Boys patients vs Boys HC); ## *p* < 0.01(Boys patients vs Boys HC); °°*p* < 0.01 (Girls patients vs Girls HC); °°°*p* < 0.001 (Girls patients vs Girls HC); §*p* < 0.05 (Boys patients vs Girls patients); §§*p* < 0.01 (Boys patients vs Girls patients). On the right typical flow cytometric measures of a representative HC boy and a representative patient boy, and a representative HC girl and a representative patient girl. (Pt = patient; HC = control group)

Figure 1a shows that the amount of CP^•^ was significantly increased (*p* < 0.01) in blood from patients with respect to sex-matched HCs.

Analyzing data by sex, CP^•^ amount was not significantly different between HC children, as well as between patients with bronchiolitis, although in these latter the CP^•^ mean value was higher in boys. These data highlighted that there is significant ROS production in the disease regardless of sex.

### Platelet activation and death

Phosphatidylserine (PS) externalization, a typical marker of early apoptosis in nucleated cells, has recently been associated with “high” platelet activation features more than with platelet apoptosis. Normally confined in the membranes inner leaflet, PS mediates platelet pro-coagulant function and regulates platelet life span when exposed on the outer membrane surface of activated platelets. It causes coagulation and thrombosis by providing a suitable surface for assembly of the pro-thrombinase complex, which converts prothrombin to thrombin [11]. In this study we found that in in HCs there are no significant differences in platelets with PS externalization between boys and girls. Interestingly, in boys the comparison between HCs and Pt is statistically significant. In particular, we note that in boys with bronchiolitis, the percentage of platelets with PS externalization (annexin V positives) was significantly higher than both HCs and girls with bronchiolitis (*p* < 0.01) (Fig. 1b**)**. Interestingly, when a double labeling (fluorescein Isothiocyanate-conjugated annexin V and trypan blue) analysis was performed, we found that the number of double positive platelets, i.e. apoptotic cells, was very low in both boys and girls with bronchiolitis and was comparable with that detected in HCs (mean values: 1.3 ± 0.5% vs 1.06 ± 0.7%).

### Platelet adhesion

Platelet adhesion was investigated by measuring the expression of surface P-selectin (or CD62) (Fig. 1c) and of integrin GP αIIbβ3 (evaluated as PAC-1 positive cells) (Fig. 1d) [9, 12].

P-selectin, stored in α-granules in the inner of resting platelets and expressed on the platelet surface after activation, is a marker of platelet adhesion, in particular of heterotypic platelet adhesion. Integrin αIIbβ3 is a fibrinogen receptor playing a vital role in platelet thrombus, because it is required for homotypic platelet adhesion [13]. We found that in girls with bronchiolitis about 35% of platelets expressed P-selectin in surface (Fig. 1c). In particular, significant differences were found between patients girls and HCs girls (*p* < 0.001).

Interestingly, analyzing the control groups significant sex differences were found in the percentage of platelets expressing P-selectin (*p* < 0.01). Compared to control boys, girls have a lower percentage of platelet P-selectin positive (16,89% vs 30,63% respectively).

This difference, already reported in the literature [14, 15], could be due to a shedding phenomenon of cell membrane.

Regarding integrin GP αIIbβ3 activation the trend is opposite to the P-selectin. In particular, integrin GP αIIbβ3 significantly increase in patient boys respect to HC (*p* < 0.05), whereas in girls with bronchiolitis significantly decrease respect to boys patients (*p* < 0.05) (Fig. 1d). Interestingly, in girls with bronchiolitis and in HCs boys the percentage of platelet PAC-1 positives was comparable (about 1%). Moreover, the data shown in Fig. 1c and d highlighted a different ability of platelets adhesion in boys and girls. In particular, a higher percentage of P-selectin positive platelets was found in HC boys with respect to HC girls, while no significant sex difference was detected in percentage of PAC-1 positive platelets.

In boys with bronchiolitis, the percentage of platelets forming homotypic aggregates increases significantly with respect to girls, while no significant differences were detected in the percentage of platelets forming heterotypic aggregates.

## Discussion

This work provides new lines of evidence showing that sex can affect the clinical course, as well as the use of a suitable therapy, of children with bronchiolitis. Sex is a significant epidemiological factor for several diseases, but its role in the development and outcome of viral infections of the respiratory tract has not been extensively studied. Literature data suggest that in bronchiolitis, infections by RSV were more frequent in boys than in girls and that boys were also more commonly hospitalized than girls [16]. Accordingly, in a selected group of patients with bronchiolitis, hospitalized between January and March 2018, we found that sex differences occurred. Compared to girls with bronchiolitis, boys showed: i) a trend to produce higher ROS levels; ii) a higher percentage of activated platelets and iii) a higher number of platelets forming homotypic aggregates (PAC-1 positives).

Increased evidences indicate that platelets can modulate host innate defenses against RSV inducing in vitro peripheral blood mononuclear cells to exert an interferon-mediated anti-RSV effect on host cells and to decrease viremia through viral particles internalization [17]. Importantly, platelets have been hypothesized to play a key role in the development of RSV-mediated secondary thrombocytosis [2, 18, 19]. RSV infection stimulated in vitro human mononuclear phagocytes to increase synthesis and release of platelets-activating factor, which could have a critical role in platelet activation, aggregation and thrombocytosis occurring [20]. Interestingly, increased RSV-linked expression of platelet-related genes have been measured in children affected by lower respiratory tract infections [21]. Our results support the hypothesis that activated platelets in blood of children affected by RSV-induced bronchiolitis could contribute, in a sex-dependent manner, to thrombocytosis. This assertion is supported by the findings that: i) platelet whit annexin V positives are highly activated, with pro-coagulant functions and whit integrin αIIbβ3 in an activated state (platelet PAC-1 positives); and ii) platelets with P-selectin on surface (forming heterotypic aggregates) play a crucial role in thrombus formation inter-connecting inflammatory and coagulation activity [5, 10]. In particular, we hypothesize that a sort of “hyper-aggregation” of platelet could determine an increased thromboembolic complication in girls with bronchiolitis precisely because of this significant production of heterotypic aggregates.

In agreement with previous results showed by Dundaroz et al., [4] our data showed an increased sex-independent pro-oxidant status in blood of children with RSV-mediated bronchiolitis. RSV has been reported to induce in vitro ROS formation and downregulation of antioxidant enzymes [22]. We hypothesized that: i) an increase of intracellular ROS could induce functional alteration in platelets, resulting in inflammation-driven disorders; and ii) as well as others studies, the male sex represents a greater risk for the severity of bronchiolitis, with a more frequent hospitalization and oxygen based treatments [23–25]. Further studies will be needed to determine the contribution of activated platelets to OxS measured in bronchiolitis.

We can however hypothesize a close link between platelet activation and OxS also in bronchiolitis. Several papers reported that these cells are critical components of inflammation-mediated pulmonary acute lung injury and that in inflamed pulmonary diseases platelets can be both sources and target of ROS [26–28]. This behavior could contribute to amplify lung injury and tissue inflammatory response as well as, in a vicious cycle, to a further platelet activation. With this regard, the use of pharmacological approaches aimed to control ROS production and to restore tissue redox imbalance could improve platelet physiology and functions as well as to limit the lung and the cardiovascular injury linked to the ROS-mediated direct- and the indirect platelet activation in this disease.

## Conclusions

RSV is a major cause of upper and lower respiratory tract infections in children, for which no specific treatment or vaccine is currently available. Modulation of OxS may represent potential novel pharmacological approaches to ameliorate RSV-induced acute lung inflammation and its long-term consequences.

In summary, we can affirm that the data obtained could help to have a different therapeutic approach if related to the clinical picture and the sex of the patient.

Moreover, we think that sex differences identified in this work should be studied in depth increasing, in addition, the number of cases analyzed.

## Data Availability

The datasets used and/or analyzed during the current study are available from the corresponding author on reasonable request.

## Abbreviations

ACD: Acid-citrate- dextrose tubes
CP: Nitroxide 3-carboxyproxyl radical
EPR: Electron para-magnetic resonance
HCs: Healthy controls subjects
OxS: Oxidative stress
PRP: Platelet-rich plasma
PS: Phosphatidylserine
ROS: Reactive oxidizing species
RSV: Respiratory syncytial virus
RSVA: Respiratory syncytial virus A
RSVB: Respiratory syncytial virus B
RT-PCR: Real-time PCR
